# Supplementary material for: Identification of Early Esophageal Cancer by Semantic Segmentation
Source: J Pers Med. 2022 Jul 25;12(8):1204. doi: 10.3390/jpm12081204 (PMC9331549; doi:10.3390/jpm12081204)
Supplement: Supplementary file 1 [file jpm-12-01204-s001.zip › jpm-1769698-supplementary.pdf]

Journal of *Personalized Medicine*

MDPI

Article

Identification of Early Esophageal Cancer by Semantic Seg-Mentation: Supplement Material

Yu-Jen Fang [1,2], Arvind Mukundan [3], Yu-Ming Tsao [3], Chien-Wei Huang [4,5,*] and Hsiang-Chen Wang [3,*]

[1] Department of Internal Medicine, National Taiwan University Hospital, Yun-Lin Branch, No. 579, Sec. 2, Yunlin Rd., Douliu City, Yunlin County 640, Taiwan; toby851072@gmail.com (Y.-J.F.)
[2] Department of Internal Medicine, National Taiwan University College of Medicine, No.1 Jen Ai Rd. Sec. 1, Taipei City 100, Taiwan
[3] Department of Mechanical Engineering, Advanced Institute of Manufacturing with High Tech Innovations (AIM-HI), Center for Innovative Research on Aging Society (CIRAS), National Chung Cheng University, 168, University Rd., Min Hsiung, Chia Yi 62102, Taiwan; d09420003@ccu.edu.tw (A.M.); tony00013@gmail.com (Y.-M.T.)
[4] Department of Gastroenterology, Kaohsiung Armed Forces General Hospital, 2, Zhongzheng 1st. Rd., Lingya District, Kaohsiung 80284, Taiwan
[5] Department of Nursing, Tajen University, 20, Weixin Rd., Yanpu Township, Pingtung 90741, Taiwan
* Correspondence: forevershiningfy@yahoo.com.tw (C.-W.H.); hcwang@ccu.edu.tw (H.-C.W.)

Abstract: This article provides the supplementary information for the article Identification of Early Esophageal Cancer by Semantic Seg-mentation. Section 1 gives a brief overview of the narrowband imaging while the second section explains about the semantic segmentation. The third section explains the encoder-decoder model used in this study and the last section gives an overview of the U-Net and ResNet152 models.

Keywords: esophageal cancer, small data, semantic segmentation, encoder-decoder model, U-Net, ResNet150V2, white light imaging; Narrow band imaging

Citation: Fang, Y.-J.; Mukundan, A.; Tsao, Y.-M.; Huang, C.-W.; Wang, H.-C. Identification of Early Esophageal Cancer by Semantic Segmentation. *J. Pers. Med.* **2022**, *12*, 1204. https://doi.org/10.3390/jpm12081204

Academic Editors: Chin-Sheng Lin, Chin Lin and Hung-Yu Wei

Received: 29 May 2022
Accepted: 22 July 2022
Published: 25 July 2022

Publisher's Note: MDPI stays neutral with regard to jurisdictional claims in published maps and institutional affiliations.

Copyright: © 2022 by the authors. Licensee MDPI, Basel, Switzerland. This article is an open access article distributed under the terms and conditions of the Creative Commons Attribution (CC BY) license (https://creativecommons.org/licenses/by/4.0/).

Narrow Band Imaging (NBI):

The traditional white light endoscope (WLI) is composed of three primary colors blue, red and green, and the principle of WLI is to irradiate the light source to the mucosal tissue, and then collect the reflection spectrum to form an image. The wavelength of light determines the penetration depth of light in the tissue. In visible light, the penetration depths of the three types of light are in sequence: red light (R) > green light (G) > blue light (B), that is, the longer the wavelength, shorter the penetration depth. The narrower band imaging technology (NBI) is an imaging technology that narrows only in the bandwidth of blue light and green light while reducing the contribution of the red-light band, using blue light (400-430nm) and green light (525- 555nm) thereby processing the subtle images of the mucosal surface to be sharper and increase the contrast [18]. The NBI system uses a xenon lamp light through a narrow-band filter to filter out narrow-band light in two wavelength bands, blue (415nm) and green (540nm). The penetration depth of visible light wavelength increases with the increase of wavelength, and the wavelength band of the red-light component is discarded, and then only the blue and green light bands are used to irradiate the tissue. In the narrowband imaging system, the microvessels of the superficial mucosal tissue are brown (415 nm band), and the blood vessels of the submucosal tissue are cyan (540 nm band), which is more layered than the WLI, which is more favorable to distinguish. The Charge-coupled Device (CCD) in the system will receive the narrow-band blue and green light reflected by the mucosa and convert them into digital signals at the same time. The blue light is distributed to the B and G channels, and the green light is distributed to the R channel, resulting in three new R, G, and B channels, so that the original endoscopic image with only blue and green components can be displayed in color on the screen [24,25].

Semantic segmentation

Several important tasks of deep learning in the field of Computer Vision (CV) are Image Classification, object detection, image segmentation. And among these, Image Segmentation is for pixel detection and classification which can be applied to tasks such as face recognition, autonomous driving, and medical image analysis. Image segmentation can be divided into Semantic Segmentation, instance segmentation, panoramic segmentation. This study uses semantic segmentation, which means to classify all pixels in the image, which can accurately mark the location of the target and automatically know its classification. Image segmentation is a new field opened by the emergence of FCN, and the field of image segmentation has been continuously developed, such as: Google portrait mode, YouTube stories, Virtual make-up, Virtual try-on, Self-driving cars, etc. There are also many models for semantic segmentation so far, such as FCN, DeconvNet, U-Net, SegNet, DeepLab, RefineNet, PSPNet, GSCNN based on convolutional neural network (CNN). There are many model bases and model variations such as ReNet and ReSeg based on Recurrent Neural Network (RNN), and this study uses U-Net based on CNN as the research architecture.

Encoder-Decoder model

Neural network is the main axis of modern technology, and recently it has developed from the original neural network and machine learning to the current deep learning, and the method used in this study is deep learning. There are many models of deep learning. So far, much research has been conducted on CNN, RNN and long short-term memory neural network (LSTM). The method used in this study is constructed with reference to the Encoder-Decoder model as shown in the figure S1. The Encoder-Decoder model is a method that uses recurrent neural networks for sequence-to-sequence prediction problems. It was originally invented for machine translation problems. This method involves two recurrent neural networks. One encodes the input sequence, called the encoder, and the other decodes the encoded input sequence into the target sequence, called the decoder. This model can be applied to chatbots, machine translation, text summary, and image captioning. However, the main limitation of simple encoder-decoder models is that all information needs to be summarized in a one-dimensional (1-D) vector, which is extremely difficult to implement for long input sequences. Understanding the Encoder-Decoder model is key to recent advances in Natural Language Processing (NLP).

U-Net and ResNet152

ResNet won the ImageNet Large Scale Visual Recognition Challenge (ILSVRC) in 2015, and its characteristic is the Residual block, which reduces the gradient vanish problem when the gradient is deep and proposes a residual learning framework. It makes it possible to train a deeper model, successfully reaching 152 layers. Residual block, as shown in Figure S2, is used to deal with gradient vanishing caused by deep neural network layers and too many parameters. Although Normalization can be used to solve it, it cannot prevent degradation. When the depth increases, the accuracy will reach saturation and drop rapidly. Figure S3 is the ResNet architecture diagram used in ImageNet. The Residual block is used at most 34 layers in the research, and the next 50/101/152 layers are modified the building block structure into a Bottleneck building block, as shown in Figure S4, reduce the 3x3 Conv computational burden through 1x1 Conv dimension reduction, and then use 1x1 Conv to increase the dimension back to the original shape. Table S1 compares the error rates of each architecture. Figure S5 shows the ResNet152V2+U-Net Architecture Diagram while Figure S6 shows the Accuracy of training results. Figures S6 (a) and (b) is the training accuracy of WLI images of the first 25 and the last 10 Epochs. Figures S6 (c) and (d) is the training accuracy of NBI images of the first 25 and the last 10 Epochs.

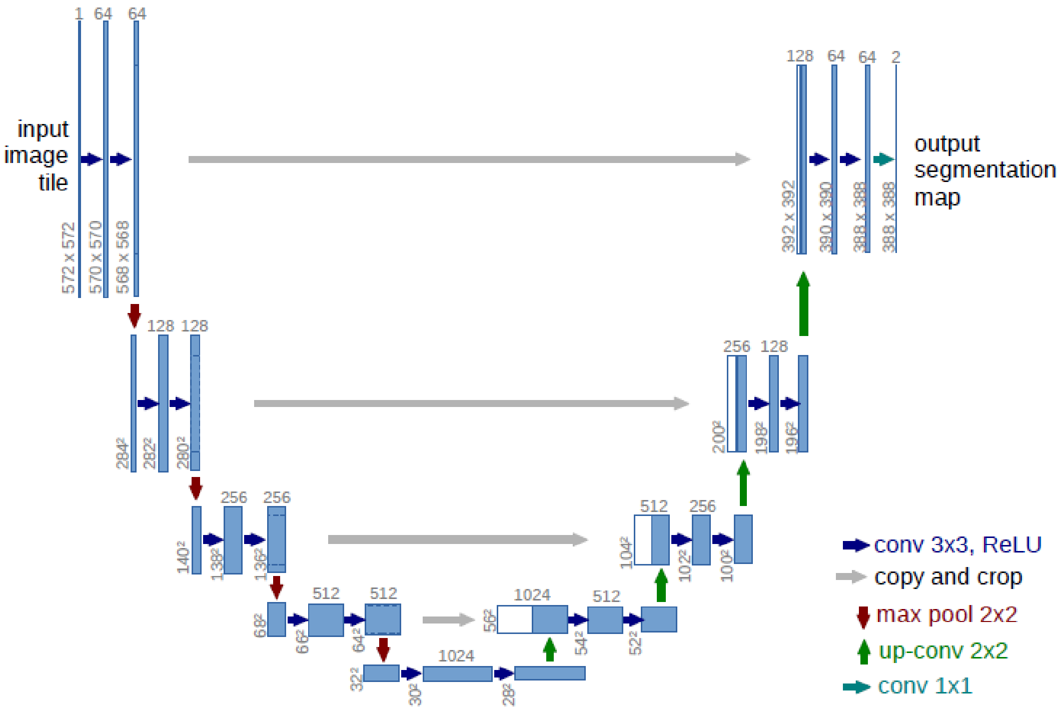

Figure S1. Encoder-Decoder simple model diagram.

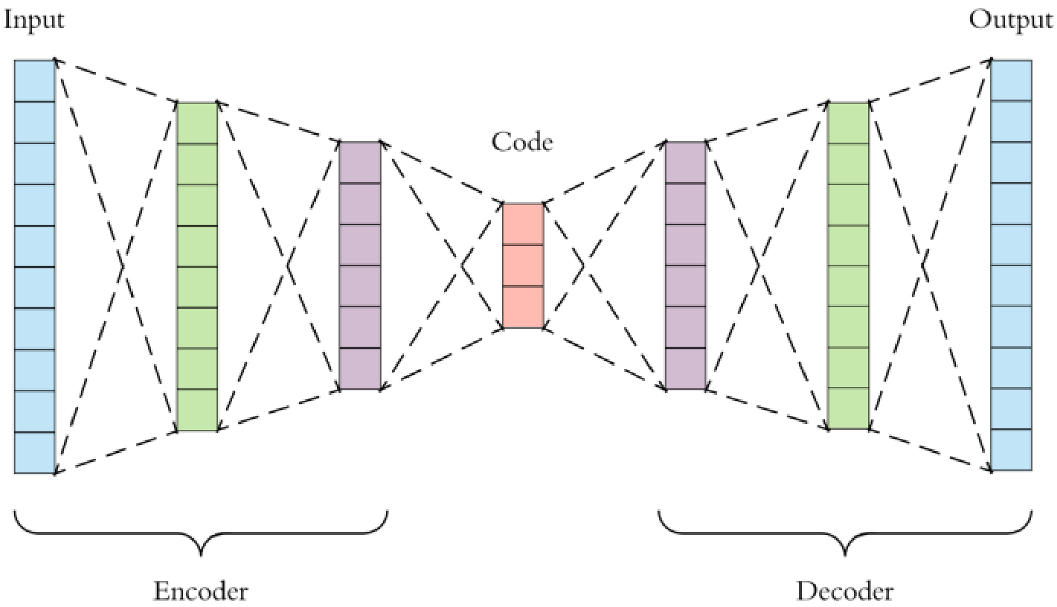

Figure S2. U-Net original architecture diagram.

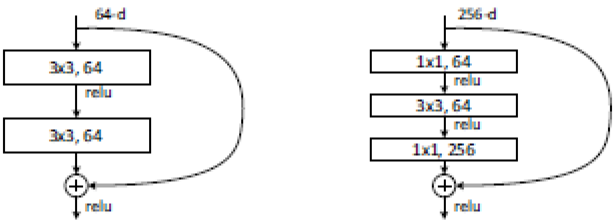

Figure S3. Residual block.

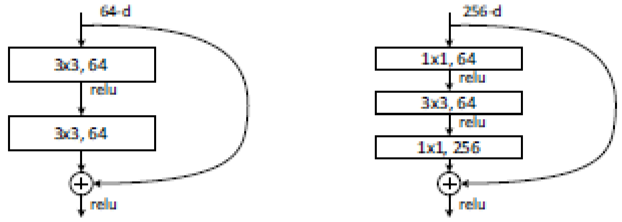

Figure S4. Bottlenet building block.

Table S1. Architecture diagram of ResNet for ImageNet.

layer name	output size	18-layer	34-layer	50-layer	101-layer	152-layer
conv1	112×112	7×7, 64, stride 2				
conv2_x	56×56	3×3 max pool, stride 2				
		[3×3, 64<br>3×3, 64] ×2	[3×3, 64<br>3×3, 64] ×3	[1×1, 64<br>3×3, 64<br>1×1, 256] ×3	[1×1, 64<br>3×3, 64<br>1×1, 256] ×3	[1×1, 64<br>3×3, 64<br>1×1, 256] ×3
conv3_x	28×28	[3×3, 128<br>3×3, 128] ×2	[3×3, 128<br>3×3, 128] ×4	[1×1, 128<br>3×3, 128<br>1×1, 512] ×4	[1×1, 128<br>3×3, 128<br>1×1, 512] ×4	[1×1, 128<br>3×3, 128<br>1×1, 512] ×8
conv4_x	14×14	[3×3, 256<br>3×3, 256] ×2	[3×3, 256<br>3×3, 256] ×6	[1×1, 256<br>3×3, 256<br>1×1, 1024] ×6	[1×1, 256<br>3×3, 256<br>1×1, 1024] ×23	[1×1, 256<br>3×3, 256<br>1×1, 1024] ×36
conv5_x	7×7	[3×3, 512<br>3×3, 512] ×2	[3×3, 512<br>3×3, 512] ×3	[1×1, 512<br>3×3, 512<br>1×1, 2048] ×3	[1×1, 512<br>3×3, 512<br>1×1, 2048] ×3	[1×1, 512<br>3×3, 512<br>1×1, 2048] ×3
	1×1	average pool, 1000-d fc, softmax				
FLOPs		1.8×10^9	3.6×10^9	3.8×10^9	7.6×10^9	11.3×10^9

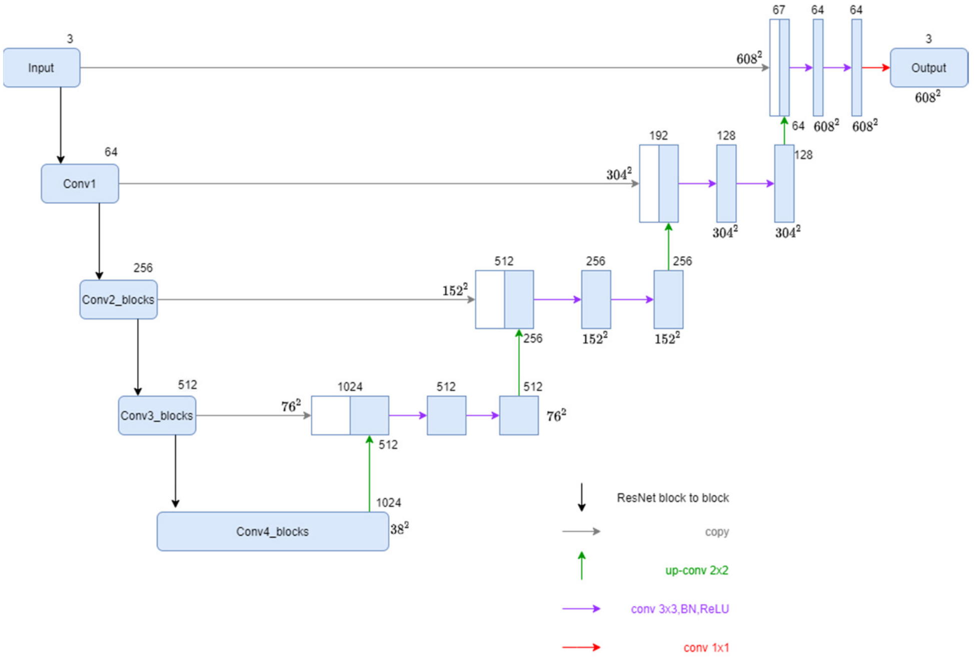

Figure S5. ResNet152V2+U-Net Architecture Diagram.
